# Crystal structure of ethyl 4-[4-(di­methyl­amino)­phen­yl]-2,7,7-trimethyl-5-oxo-1,4,5,6,7,8-hexa­hydro­quinoline-3-carboxyl­ate

**DOI:** 10.1107/S2056989018011982

**Published:** 2018-09-11

**Authors:** Scott A. Steiger, Chun Li, Nicholas R. Natale

**Affiliations:** a Department of Biomedical and Pharmaceutical Sciences, The University of Montana, 32 Campus Drive, Missoula, MT 59812, USA; b Department of Chemistry, Ithaca College, 953 Danby Road, Ithaca, NY 14850, USA

**Keywords:** crystal structure, structure-activity relationships, calcium-channel antagonists, 4-aryl-hexa­hydro­quinolines, 1,4-di­hydro­pyridine rings, hydrogen bonding

## Abstract

In the title racemic compound, ethyl 4-(4-di­methyl­amino­phen­yl)-2,7,7-trimethyl-5-oxo-1,4,5,6,7,8-hexa­hydro­quinoline-3-carboxyl­ate, the common structural features in this type of compound, such as the flat-boat conformation of the 1,4-di­hydro­pyridine (1,4-DHP) ring, the envelope conformation of the fused cyclo­hexa­none, and the substituted phenyl ring at the pseudo-axial position and orthogonal to the 1,4-DHP ring, are present. In the crystal, mol­ecules are linked *via* N—H⋯O and C—H⋯O hydrogen bonds, forming layers parallel to the (10) plane.

## Abstract 2

In the title racemic compound, the 1,4-di­hydro­pyridine (1,4-DHP) ring adopts a flat-boat conformation, which is bis­ected by the plane of the pseudo-axial aryl ring, while the cyclo­hexa­none ring adopts an envelope conformation. In the crystal, mol­ecules are linked *via* N—H⋯O and C—H⋯O hydrogen bonds, forming layers parallel to (10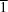).

## Chemical context   

1,4-Di­hydro­pyridine (DHP) derivatives are well known for their calcium-channel blocking activity and many of these compounds, such as nifedipine, nicardipine, and amlodipine, have been used in the treatment of angina pectoris and systemic hypertension. (Wishart *et al.*, 2006 ▸) 4-Aryl-1,4-di­hydro­pyridines that bind the *L*-type voltage-gated calcium channels (VGCC) have been in general medical practice for over three decades (Zamponi, 2005 ▸). Many modifications on 1,4-DHP have been performed to obtain active compounds as calcium-channel agonists or antagonists (Martín *et al.*, 1995 ▸; Rose, 1990 ▸; Rose & Dräger 1992 ▸). One such modifications is fusing a cyclo­hexa­none ring to form hexa­hydro­quinolone, in which the orientation of the carbonyl group of the ester substituent at the 5-position in the 1,4-DHP ring has been fixed. This class of compounds has been shown to have moderate calcium-channel antagonistic activity, as well as anti-inflammatory modes and stem-cell differentiation properties, and has been implicated in slowing neurodegenerative disorders (Trippier *et al.*, 2013 ▸). Recently, these compounds were found to have distinct selectivity profiles to different calcium channel subtypes (Schaller *et al.*, 2018 ▸). Another report also showed that the 4-aryl-hexa­hydro­quinolones, especially the ones containing a meth­oxy moiety, exhibit good anti­oxidant property as radical scavengers (Yang *et al.*, 2011 ▸). It has been revealed that the aryl group in the 4-position of the 1,4-DHP ring is the basic requirement for optimal activity and the type of electron-withdrawing groups on the phenyl group would affect the receptor-binding activity (Takahashi *et al.*, 2008 ▸). It has also been proven that the flattened boat conformation of the 1,4-DHP ring is one factor that leads to higher calcium-channel activity (Linden *et al.*, 2004 ▸). In a continuation of our study on the structure–activity relationship of this class of 1,4-DHP derivatives, *i.e.* 4-aryl-hexa­hydro­quinolones, we report herein the crystal structure of a compound we synthesized, ethyl 4-(4-di­methyl­amino­phen­yl)-2,7,7-trimethyl-5-oxo-1,4,5,6,7,8- hexa­hydro­quinoline-3-carboxyl­ate.
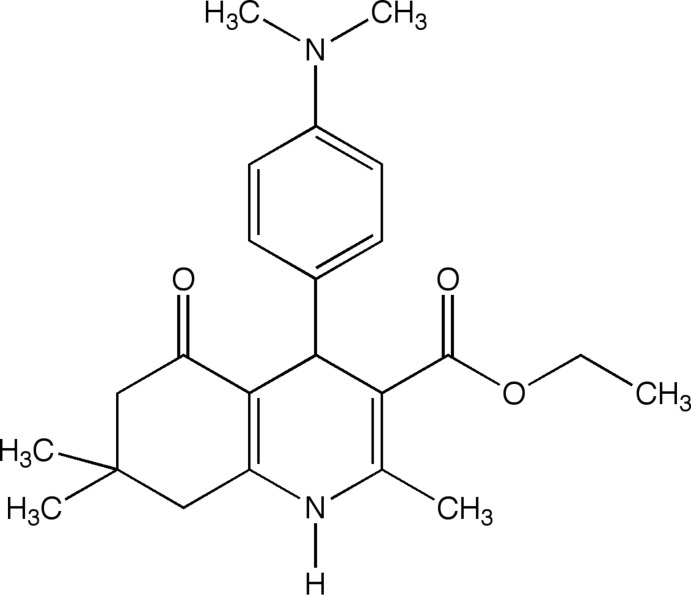


## Structural commentary   

**Figure 1:**
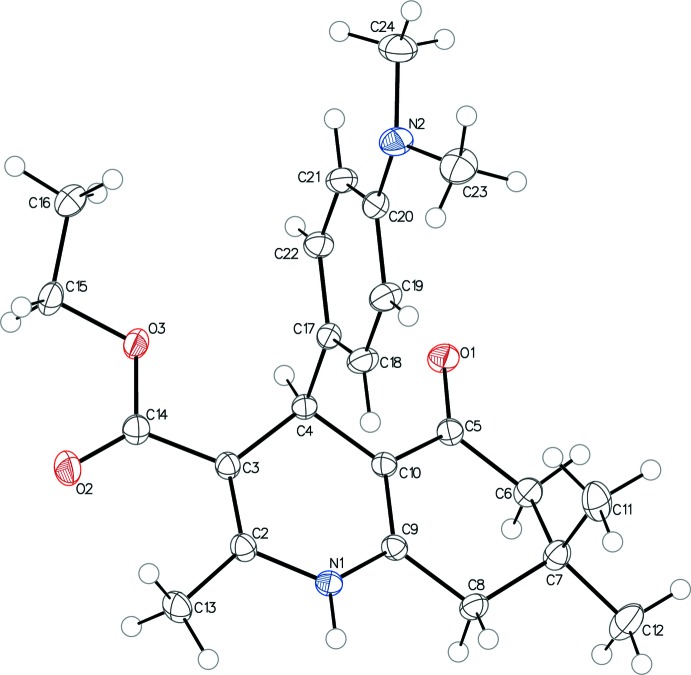
The asymmetric unit of the title compound showing the atom-labeling scheme. Displacement ellipsoids are drawn at the 50% probability level

The asymmetric unit of the title compound contains one independent mol­ecule crystallizing racemically in the monoclinic space group *P*2_1_ */n*. A displacement ellipsoid plot showing the atomic numbering is presented in Fig. 1 ▸.

In the title compound, the 1,4-DHP ring is characterized by a shallow or flattened boat conformation, which is one of the factors that leads to higher calcium-channel activity. The flattened boat conformation is visually obvious with the flat base formed by atoms C2, C3, C10, and C9 and the bow and stern formed by the slightly raised C4 and N1 atoms. The mean plane defined by atoms C2, C3, C9, and C10 is planar, with an r.m.s. deviation of 0.008 Å. The shallowness of the boat conformation is indicated by the marginal displacements of atoms N1 [0.1332 (15) Å] and C4 [0.3047 (16) Å] from this mean plane, and is also implied by the small puckering amplitude *Q* [0.2583 (10) Å].

Examination of the fused C5–C10 cyclo­hexa­none ring using puckering parameters also showed that the ring adopts an envelope conformation, with atom C7 protruding in the same direction as the 4-aryl group at a distance of 0.6453 (15) Å from the mean plane through the other five C atoms. The orientation of ring atom C7 makes the axial bond C7—C11 *syn*-periplanar to the 4-aryl group, *i.e.* the torsion angle between C7—C11 and C4—C17 is 6.91 (7)°.

The pseudo-axial position of the 4-aryl group is conserved in the title compound. The C17–C22 phenyl ring is almost orthogonal to the base of the 1,4-DHP ring formed by atoms C2, C3, C10, and C9, with the dihedral angles between the C2–C3–C10–C9 mean plane and the ring being 89.59 (3)°. The very small pseudo-torsion angle [2.44 (8)°] between N1—C4 and C17—C18 also implies a bis­ecting orientation of the 4-aryl group to the 1,4-DHP ring in this compound.

As in other 1,4-DHP compounds (Linden *et al.*, 2004 ▸), the ester group is coplanar and at a *cis* orientation to the adjacent endocyclic C2=C3 double bond. The planarity extends out through the ester chains.

The nitro­gen atom in the di­methyl­amino group is almost in the same plane as the phenyl ring, at a distance of 0.0420 (16) Å from the mean plane. However, the plane formed by N2–C23–C24 is slightly bent from the phenyl group with the angle of 27.24 (11)° rather than being coplanar with the phenyl ring, which seems to be common in *N*,*N*-di­methyl­aniline type of compounds (Dahl, 2000 ▸). In conclusion, the parameters reported here demonstrate that the conformational features usually observed in cyclo­hexa­none-fused 1,4-DHP derivatives have been conserved. As a promising base structure for calcium-channel antagonists, different substitutions and more structural modifications are being carried out in our group. Progress will be reported in due course.

## Supra­molecular features   

**Figure 2:**
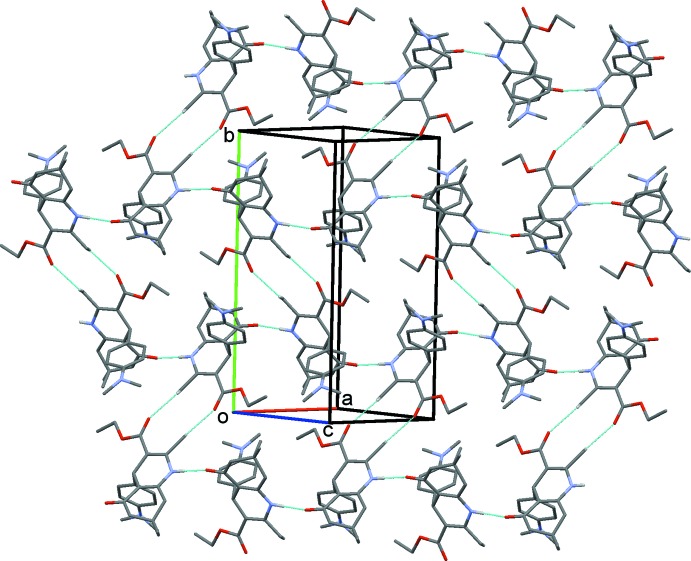
A view normal to plane (10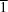) of the crystal packing of the title compound. The hydrogen bonds (Table 1 ▸) are shown as dashed lines and only H atoms H1 and H13*C* have been included.

In the crystal, mol­ecules are linked along the diagonal of the *ac* plane by N—H⋯O hydrogen bonds, forming chains which are in turn linked by C—H⋯O hydrogen bonds into layers parallel to the (10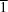) plane ­(see Fig. 2 ▸ and Table 1 ▸).

## Database survey   

A search in the Cambridge Structural Database (Version 5.39, November 2017) for related compounds with a 4-aryl-hexa­hydro­quinolone-3-carboxyl­ate fragment gave 30 hits. All these compounds share the common structural features such as the flat-boat conformation of the1,4-di­hydro­pyridine (1,4-DHP) ring, the envelope conformation of the fused cyclo­hexa­none ring, and the substituted phenyl ring at the pseudo-axial position and orthogonal to the 1,4-DHP ring.

## Synthesis and crystallization   

An oven-dried 100 ml round-bottom flask equipped with a magnetic stir bar was charged with 10 mmol of dimedone, 10 mmol of ethyl aceto­acetate and 5 mol % of ytterbium(III) tri­fluoro­methane­sulfonate. The mixture was then taken up in 30 ml of absolute ethanol, capped and put under an inert atmosphere of argon, after which the solution was allowed to stir at room temperature for 20 min. The appropriate corresponding benzaldehyde (10 mmol) and 10 mmol of ammonium acetate were added to the stirring solution, the solution was allowed to stir at room temperature for 48 h. Reaction progress was monitored *via* TLC. Once the reaction was complete, excess solvent was removed *via* rotary evaporation. The solution was then purified *via* silica column chromatography. The title compound was recrystallized by slow evaporation from hexane and ethyl acetate (*v*:*v* = ?:?). m.p. 513.1 K.

^1^H NMR (δ, CDCl_3_) p.p.m. 0.95 (*s*, 3H), 1.05 (*s*, 3H), 1.22 (*t*, *J* = 7.08 Hz, 3H), 2.13 & 2.19 (*ABq*, 2H, *J* = 16.5 Hz), 2.17 & 2.27 (*ABq*, 2H, *J* = 16.5 Hz), 2.85 (*s*, 3H), 4.046 (*q*, *J* = 7.08 Hz, 2H), 4.94 (*s*, 1H), 6.06 (*s*, 1H), 6.57 (*d*, *J* = 6.8 Hz, 2H), 7.14 (*d*, *J* = 6.8 Hz, 2H).

^13^C NMR (δ, CDCl_3_) p.p.m. 14.37, 19.55, 27.42, 29.54, 32.82, 35.38, 40.86, 41.17, 50.85, 59.84, 106.61, 112.46, 128.70, 135.94, 142.95, 147.85, 149.06, 167.79, 195.75.

MS: calculated for C_23_H_30_N_2_O_3_, 382.49; observed**:** *m*/*z* = 405 ([*M* + 23] 7), 234 (100).

## Refinement   

Crystal data, data collection and structure refinement details are summarized in Table 2 ▸. The H atoms on methyl groups were constrained to an ideal geometry, with C—H = 0.98 Å and *U*_iso_(H) = 1.5*U*_eq_(C), and were allowed to rotate freely about the C—C bonds. The rest of the H atoms were placed in calculated positions with C—H = 0.95–1.00 Å and refined as riding on their carrier atoms with *U*_iso_(H) = 1.2*U*_eq_(C). The positions of the amine H atoms and hydroxyl H atoms were determined from difference-Fourier maps and freely refined. Three low-angle reflections were omitted from the refinement because their observed intensities were much lower than the calculated values as a result of being partially obscured by the beam stop.

## Supplementary Material

Crystal structure: contains datablock(s) I. DOI: 10.1107/S2056989018011982/ds2251sup1.cif

Structure factors: contains datablock(s) I. DOI: 10.1107/S2056989018011982/ds2251Isup2.hkl

Supporting information file. DOI: 10.1107/S2056989018011982/ds2251sup2.cdx

Supporting information file. DOI: 10.1107/S2056989018011982/ds2251Isup4.cml

CCDC reference: 1863662

Additional supporting information:  crystallographic information; 3D view; checkCIF report

## Figures and Tables

**Table 1:** Hydrogen-bond geometry (Å, °)

*D*—H⋯*A*	*D*—H	H⋯*A*	*D*⋯*A*	*D*—H⋯*A*
N1—H1⋯O1^i^	0.879 (15)	1.968 (15)	2.8380 (11)	170.6 (14)
C13—H13*C*⋯O2^ii^	0.98	2.57	3.1741 (15)	120

Symmetry codes: (i) 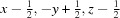; (ii) 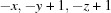.

**Table 2:** Experimental details

Crystal data	
Chemical formula	C_23_H_30_N_2_O_3_
*M*_r_	382.49
Crystal system, space group	Monoclinic, *P*2_1_/*n*
Temperature (K)	100
*a*, *b*, *c* (Å)	9.6834 (3), 18.2390 (5), 12.2073 (3)
β (°)	105.4464 (14)
*V* (Å^3^)	2078.12 (10)
*Z*	4
Radiation type	Mo *K*α
μ (mm^−1^)	0.08
Crystal size (mm)	0.46 × 0.30 × 0.26

Data collection	
Diffractometer	Bruker SMART BREEZE CCD
Absorption correction	Multi-scan (*SADABS*; Bruker, 2013 ▸)
*T*_min_, *T*_max_	0.923, 1.000
No. of measured, independent and observed [*I* > 2σ(*I*)] reflections	48649, 7211, 5703
*R*_int_	0.038
(sin θ/λ)_max_ (Å^−1^)	0.747

Refinement	
*R*[*F* ^2^ > 2σ(*F* ^2^)], *wR*(*F* ^2^), *S*	0.049, 0.142, 1.02
No. of reflections	7211
No. of parameters	264
H-atom treatment	H atoms treated by a mixture of independent and constrained refinement
Δρ_max_, Δρ_min_ (e Å^−3^)	0.56, −0.21

Computer programs: *APEX2* and *SAINT* (Bruker, 2013 ▸), *SHELXS97* (Sheldrick, 2008 ▸), *SHELXL2014* (Sheldrick, 2015 ▸) and *OLEX2* (Dolomanov *et al.*, 2009 ▸).

## supplementary crystallographic information

### Crystal data

**Table:**

C_23_H_30_N_2_O_3_	*D*_x_ = 1.223 Mg m^−^^3^
*M**_r_* = 382.49	Melting point: 240.1 K
Monoclinic, *P*2_1_/*n*	Mo *K*α radiation, λ = 0.71073 Å
*a* = 9.6834 (3) Å	Cell parameters from 9940 reflections
*b* = 18.2390 (5) Å	θ = 2.5–31.8°
*c* = 12.2073 (3) Å	µ = 0.08 mm^−^^1^
β = 105.4464 (14)°	*T* = 100 K
*V* = 2078.12 (10) Å^3^	Prism, yellow
*Z* = 4	0.46 × 0.30 × 0.26 mm
*F*(000) = 824	

### Data collection

**Table:**

Bruker SMART BREEZE CCD diffractometer	5703 reflections with *I* > 2σ(*I*)
Radiation source: 2 kW sealed X-ray tube	*R*_int_ = 0.038
φ and ω scans	θ_max_ = 32.1°, θ_min_ = 2.4°
Absorption correction: multi-scan (SADABS; Bruker, 2013)	*h* = −13→14
*T*_min_ = 0.923, *T*_max_ = 1.000	*k* = −27→27
48649 measured reflections	*l* = −18→18
7211 independent reflections	

### Refinement

**Table:**

Refinement on *F*^2^	Primary atom site location: structure-invariant direct methods
Least-squares matrix: full	Hydrogen site location: mixed
*R*[*F*^2^ > 2σ(*F*^2^)] = 0.049	H atoms treated by a mixture of independent and constrained refinement
*wR*(*F*^2^) = 0.142	*w* = 1/[σ^2^(*F*_o_^2^) + (0.0858*P*)^2^ + 0.4034*P*] where *P* = (*F*_o_^2^ + 2*F*_c_^2^)/3
*S* = 1.02	(Δ/σ)_max_ < 0.001
7211 reflections	Δρ_max_ = 0.56 e Å^−^^3^
264 parameters	Δρ_min_ = −0.21 e Å^−^^3^
0 restraints	

### Special details

**Table:**

Geometry. All esds (except the esd in the dihedral angle between two l.s. planes) are estimated using the full covariance matrix. The cell esds are taken into account individually in the estimation of esds in distances, angles and torsion angles; correlations between esds in cell parameters are only used when they are defined by crystal symmetry. An approximate (isotropic) treatment of cell esds is used for estimating esds involving l.s. planes.

### Fractional atomic coordinates and isotropic or equivalent isotropic displacement parameters (Å^2^)

**Table:**

	*x*	*y*	*z*	*U*_iso_*/*U*_eq_	
O1	0.56627 (8)	0.18519 (4)	0.65486 (6)	0.02329 (16)	
O2	0.20382 (10)	0.48113 (4)	0.62250 (7)	0.0315 (2)	
O3	0.32826 (8)	0.40562 (4)	0.75768 (6)	0.02233 (16)	
N1	0.18215 (9)	0.30161 (5)	0.39338 (7)	0.01757 (16)	
N2	0.02043 (10)	0.12538 (5)	0.88241 (7)	0.02398 (19)	
C2	0.17423 (10)	0.35953 (5)	0.46481 (8)	0.01733 (18)	
C3	0.25415 (10)	0.35787 (5)	0.57458 (8)	0.01643 (17)	
C4	0.33616 (10)	0.28901 (5)	0.62517 (7)	0.01576 (17)	
H4	0.4296	0.3045	0.6776	0.019*	
C5	0.48613 (10)	0.19197 (5)	0.55790 (8)	0.01629 (17)	
C6	0.51589 (10)	0.14782 (6)	0.46162 (8)	0.01877 (18)	
H6A	0.5626	0.1012	0.4928	0.023*	
H6B	0.5842	0.1754	0.4297	0.023*	
C7	0.38262 (11)	0.13022 (6)	0.36546 (8)	0.01950 (19)	
C8	0.30521 (11)	0.20264 (6)	0.32603 (8)	0.01878 (18)	
H8A	0.3603	0.2306	0.2824	0.023*	
H8B	0.2098	0.1919	0.2744	0.023*	
C9	0.28658 (10)	0.24911 (5)	0.42229 (8)	0.01549 (17)	
C10	0.36785 (10)	0.24286 (5)	0.53149 (7)	0.01521 (17)	
C11	0.28490 (13)	0.07753 (6)	0.40759 (11)	0.0284 (2)	
H11A	0.2589	0.0993	0.4727	0.043*	
H11B	0.3353	0.0311	0.4306	0.043*	
H11C	0.1979	0.0684	0.3464	0.043*	
C12	0.42649 (14)	0.09541 (8)	0.26599 (10)	0.0327 (3)	
H12A	0.4741	0.0484	0.2901	0.049*	
H12B	0.4924	0.1282	0.2412	0.049*	
H12C	0.3411	0.0872	0.2029	0.049*	
C13	0.07190 (12)	0.41868 (6)	0.40825 (9)	0.0234 (2)	
H13A	0.0221	0.4034	0.3309	0.035*	
H13B	0.1252	0.4640	0.4053	0.035*	
H13C	0.0017	0.4273	0.4517	0.035*	
C14	0.25628 (11)	0.42135 (5)	0.64921 (8)	0.01880 (18)	
C15	0.32585 (12)	0.46169 (6)	0.84080 (9)	0.0225 (2)	
H15A	0.2268	0.4792	0.8323	0.027*	
H15B	0.3860	0.5039	0.8316	0.027*	
C16	0.38454 (12)	0.42620 (6)	0.95533 (9)	0.0232 (2)	
H16A	0.3267	0.3831	0.9614	0.035*	
H16B	0.3811	0.4613	1.0153	0.035*	
H16C	0.4840	0.4112	0.9637	0.035*	
C17	0.25404 (10)	0.24552 (5)	0.69388 (8)	0.01645 (17)	
C18	0.12125 (11)	0.21446 (6)	0.64193 (8)	0.02084 (19)	
H18	0.0818	0.2208	0.5625	0.025*	
C19	0.04507 (12)	0.17457 (6)	0.70303 (8)	0.0224 (2)	
H19	−0.0452	0.1543	0.6648	0.027*	
C20	0.09936 (11)	0.16371 (5)	0.82071 (8)	0.01958 (19)	
C21	0.23303 (11)	0.19489 (6)	0.87312 (8)	0.0213 (2)	
H21	0.2733	0.1884	0.9524	0.026*	
C22	0.30727 (11)	0.23512 (6)	0.81049 (8)	0.01973 (19)	
H22	0.3970	0.2561	0.8484	0.024*	
C23	−0.08672 (14)	0.07366 (7)	0.82229 (10)	0.0311 (2)	
H23A	−0.1338 (9)	0.0513 (5)	0.8755 (5)	0.047*	
H23B	−0.0408 (5)	0.0356 (5)	0.7881 (8)	0.047*	
H23C	−0.1578 (9)	0.0993 (3)	0.7628 (8)	0.047*	
C24	0.09169 (14)	0.10629 (7)	0.99913 (9)	0.0295 (2)	
H24A	0.0239	0.0812	1.0333	0.044*	
H24B	0.1265	0.1510	1.0422	0.044*	
H24C	0.1728	0.0738	1.0007	0.044*	
H1	0.1367 (16)	0.3063 (8)	0.3211 (13)	0.029 (4)*	

### Atomic displacement parameters (Å^2^)

**Table:**

	*U*^11^	*U*^22^	*U*^33^	*U*^12^	*U*^13^	*U*^23^
O1	0.0214 (3)	0.0261 (4)	0.0175 (3)	0.0058 (3)	−0.0032 (3)	−0.0014 (3)
O2	0.0422 (5)	0.0195 (4)	0.0272 (4)	0.0107 (3)	−0.0006 (3)	−0.0023 (3)
O3	0.0275 (4)	0.0178 (3)	0.0185 (3)	0.0034 (3)	0.0006 (3)	−0.0048 (2)
N1	0.0173 (4)	0.0179 (4)	0.0144 (3)	0.0033 (3)	−0.0012 (3)	0.0002 (3)
N2	0.0277 (5)	0.0273 (5)	0.0197 (4)	0.0008 (4)	0.0111 (3)	0.0017 (3)
C2	0.0162 (4)	0.0156 (4)	0.0188 (4)	0.0010 (3)	0.0021 (3)	0.0005 (3)
C3	0.0162 (4)	0.0149 (4)	0.0170 (4)	0.0013 (3)	0.0024 (3)	−0.0007 (3)
C4	0.0160 (4)	0.0153 (4)	0.0139 (4)	0.0011 (3)	0.0004 (3)	−0.0007 (3)
C5	0.0149 (4)	0.0160 (4)	0.0166 (4)	0.0001 (3)	0.0019 (3)	−0.0002 (3)
C6	0.0150 (4)	0.0217 (4)	0.0185 (4)	0.0032 (3)	0.0024 (3)	−0.0015 (3)
C7	0.0174 (4)	0.0210 (4)	0.0184 (4)	0.0027 (3)	0.0019 (3)	−0.0040 (3)
C8	0.0191 (4)	0.0215 (4)	0.0141 (4)	0.0026 (3)	0.0016 (3)	−0.0018 (3)
C9	0.0146 (4)	0.0156 (4)	0.0153 (4)	0.0004 (3)	0.0023 (3)	0.0008 (3)
C10	0.0154 (4)	0.0147 (4)	0.0143 (4)	0.0006 (3)	0.0019 (3)	0.0004 (3)
C11	0.0264 (5)	0.0193 (5)	0.0365 (6)	−0.0039 (4)	0.0034 (4)	−0.0031 (4)
C12	0.0304 (6)	0.0408 (7)	0.0234 (5)	0.0129 (5)	0.0013 (4)	−0.0119 (4)
C13	0.0216 (5)	0.0202 (5)	0.0244 (5)	0.0064 (4)	−0.0008 (4)	0.0019 (4)
C14	0.0173 (4)	0.0180 (4)	0.0199 (4)	0.0006 (3)	0.0027 (3)	−0.0015 (3)
C15	0.0243 (5)	0.0191 (4)	0.0227 (4)	0.0003 (4)	0.0036 (4)	−0.0073 (3)
C16	0.0222 (5)	0.0260 (5)	0.0215 (4)	−0.0034 (4)	0.0059 (4)	−0.0051 (4)
C17	0.0186 (4)	0.0153 (4)	0.0146 (4)	0.0022 (3)	0.0028 (3)	−0.0015 (3)
C18	0.0236 (5)	0.0236 (5)	0.0140 (4)	−0.0035 (4)	0.0028 (3)	−0.0013 (3)
C19	0.0242 (5)	0.0256 (5)	0.0174 (4)	−0.0043 (4)	0.0055 (4)	−0.0021 (3)
C20	0.0242 (5)	0.0181 (4)	0.0181 (4)	0.0033 (4)	0.0087 (4)	−0.0001 (3)
C21	0.0237 (5)	0.0252 (5)	0.0143 (4)	0.0044 (4)	0.0037 (3)	0.0016 (3)
C22	0.0192 (4)	0.0220 (5)	0.0157 (4)	0.0026 (3)	0.0007 (3)	0.0001 (3)
C23	0.0338 (6)	0.0328 (6)	0.0318 (6)	−0.0069 (5)	0.0173 (5)	−0.0015 (4)
C24	0.0344 (6)	0.0346 (6)	0.0225 (5)	0.0059 (5)	0.0129 (4)	0.0078 (4)

### Geometric parameters (Å, º)

**Table:**

O1—C5	1.2367 (11)	C11—H11B	0.9800
O2—C14	1.2103 (12)	C11—H11C	0.9800
O3—C14	1.3527 (12)	C12—H12A	0.9800
O3—C15	1.4451 (12)	C12—H12B	0.9800
N1—C2	1.3849 (12)	C12—H12C	0.9800
N1—C9	1.3690 (12)	C13—H13A	0.9800
N1—H1	0.879 (15)	C13—H13B	0.9800
N2—C20	1.3953 (13)	C13—H13C	0.9800
N2—C23	1.4489 (16)	C15—H15A	0.9900
N2—C24	1.4505 (14)	C15—H15B	0.9900
C2—C3	1.3575 (12)	C15—C16	1.5078 (15)
C2—C13	1.5028 (13)	C16—H16A	0.9800
C3—C4	1.5259 (13)	C16—H16B	0.9800
C3—C14	1.4700 (13)	C16—H16C	0.9800
C4—H4	1.0000	C17—C18	1.3936 (14)
C4—C10	1.5157 (13)	C17—C22	1.3917 (13)
C4—C17	1.5233 (13)	C18—H18	0.9500
C5—C6	1.5143 (13)	C18—C19	1.3867 (14)
C5—C10	1.4425 (13)	C19—H19	0.9500
C6—H6A	0.9900	C19—C20	1.4061 (14)
C6—H6B	0.9900	C20—C21	1.4024 (15)
C6—C7	1.5296 (13)	C21—H21	0.9500
C7—C8	1.5317 (14)	C21—C22	1.3903 (14)
C7—C11	1.5306 (16)	C22—H22	0.9500
C7—C12	1.5272 (15)	C23—H23A	0.976 (10)
C8—H8A	0.9900	C23—H23B	0.976 (10)
C8—H8B	0.9900	C23—H23C	0.976 (10)
C8—C9	1.4982 (13)	C24—H24A	0.9800
C9—C10	1.3603 (12)	C24—H24B	0.9800
C11—H11A	0.9800	C24—H24C	0.9800
			
C14—O3—C15	115.89 (8)	H12A—C12—H12B	109.5
C2—N1—H1	117.4 (10)	H12A—C12—H12C	109.5
C9—N1—C2	122.15 (8)	H12B—C12—H12C	109.5
C9—N1—H1	117.7 (10)	C2—C13—H13A	109.5
C20—N2—C23	118.30 (9)	C2—C13—H13B	109.5
C20—N2—C24	117.70 (10)	C2—C13—H13C	109.5
C23—N2—C24	115.49 (9)	H13A—C13—H13B	109.5
N1—C2—C13	113.49 (8)	H13A—C13—H13C	109.5
C3—C2—N1	119.47 (8)	H13B—C13—H13C	109.5
C3—C2—C13	127.03 (9)	O2—C14—O3	121.65 (9)
C2—C3—C4	121.07 (8)	O2—C14—C3	127.36 (9)
C2—C3—C14	120.30 (9)	O3—C14—C3	110.99 (8)
C14—C3—C4	118.53 (8)	O3—C15—H15A	110.5
C3—C4—H4	108.1	O3—C15—H15B	110.5
C10—C4—C3	109.87 (7)	O3—C15—C16	105.94 (8)
C10—C4—H4	108.1	H15A—C15—H15B	108.7
C10—C4—C17	111.50 (7)	C16—C15—H15A	110.5
C17—C4—C3	111.08 (8)	C16—C15—H15B	110.5
C17—C4—H4	108.1	C15—C16—H16A	109.5
O1—C5—C6	119.28 (8)	C15—C16—H16B	109.5
O1—C5—C10	122.46 (9)	C15—C16—H16C	109.5
C10—C5—C6	118.20 (8)	H16A—C16—H16B	109.5
C5—C6—H6A	108.7	H16A—C16—H16C	109.5
C5—C6—H6B	108.7	H16B—C16—H16C	109.5
C5—C6—C7	114.28 (8)	C18—C17—C4	120.98 (8)
H6A—C6—H6B	107.6	C22—C17—C4	121.92 (9)
C7—C6—H6A	108.7	C22—C17—C18	117.10 (9)
C7—C6—H6B	108.7	C17—C18—H18	119.1
C6—C7—C8	107.60 (8)	C19—C18—C17	121.80 (9)
C6—C7—C11	110.16 (9)	C19—C18—H18	119.1
C11—C7—C8	110.63 (9)	C18—C19—H19	119.5
C12—C7—C6	109.94 (9)	C18—C19—C20	121.05 (10)
C12—C7—C8	108.97 (9)	C20—C19—H19	119.5
C12—C7—C11	109.50 (9)	N2—C20—C19	120.83 (10)
C7—C8—H8A	109.0	N2—C20—C21	121.91 (9)
C7—C8—H8B	109.0	C21—C20—C19	117.22 (9)
H8A—C8—H8B	107.8	C20—C21—H21	119.6
C9—C8—C7	113.11 (8)	C22—C21—C20	120.85 (9)
C9—C8—H8A	109.0	C22—C21—H21	119.6
C9—C8—H8B	109.0	C17—C22—H22	119.0
N1—C9—C8	115.37 (8)	C21—C22—C17	121.97 (9)
C10—C9—N1	120.40 (8)	C21—C22—H22	119.0
C10—C9—C8	124.20 (8)	N2—C23—H23A	109.5
C5—C10—C4	119.80 (8)	N2—C23—H23B	109.5
C9—C10—C4	120.82 (8)	N2—C23—H23C	109.5
C9—C10—C5	119.38 (8)	H23A—C23—H23B	109.5
C7—C11—H11A	109.5	H23A—C23—H23C	109.4
C7—C11—H11B	109.5	H23B—C23—H23C	109.5
C7—C11—H11C	109.5	N2—C24—H24A	109.5
H11A—C11—H11B	109.5	N2—C24—H24B	109.5
H11A—C11—H11C	109.5	N2—C24—H24C	109.5
H11B—C11—H11C	109.5	H24A—C24—H24B	109.5
C7—C12—H12A	109.5	H24A—C24—H24C	109.5
C7—C12—H12B	109.5	H24B—C24—H24C	109.5
C7—C12—H12C	109.5		
			
O1—C5—C6—C7	151.47 (9)	C8—C9—C10—C4	−176.32 (9)
O1—C5—C10—C4	−1.56 (14)	C8—C9—C10—C5	4.11 (14)
O1—C5—C10—C9	178.01 (9)	C9—N1—C2—C3	−12.50 (14)
N1—C2—C3—C4	−8.68 (14)	C9—N1—C2—C13	168.16 (9)
N1—C2—C3—C14	175.02 (9)	C10—C4—C17—C18	−59.06 (11)
N1—C9—C10—C4	5.51 (14)	C10—C4—C17—C22	121.10 (10)
N1—C9—C10—C5	−174.06 (9)	C10—C5—C6—C7	−31.05 (12)
N2—C20—C21—C22	−177.63 (9)	C11—C7—C8—C9	72.35 (11)
C2—N1—C9—C8	−164.12 (9)	C12—C7—C8—C9	−167.21 (9)
C2—N1—C9—C10	14.20 (14)	C13—C2—C3—C4	170.56 (10)
C2—C3—C4—C10	24.82 (12)	C13—C2—C3—C14	−5.74 (16)
C2—C3—C4—C17	−99.03 (10)	C14—O3—C15—C16	168.44 (9)
C2—C3—C14—O2	−7.65 (17)	C14—C3—C4—C10	−158.81 (8)
C2—C3—C14—O3	172.85 (9)	C14—C3—C4—C17	77.33 (11)
C3—C4—C10—C5	156.41 (8)	C15—O3—C14—O2	6.08 (15)
C3—C4—C10—C9	−23.16 (12)	C15—O3—C14—C3	−174.38 (8)
C3—C4—C17—C18	63.85 (11)	C17—C4—C10—C5	−79.98 (10)
C3—C4—C17—C22	−115.98 (10)	C17—C4—C10—C9	100.45 (10)
C4—C3—C14—O2	175.96 (11)	C17—C18—C19—C20	−0.10 (16)
C4—C3—C14—O3	−3.55 (12)	C18—C17—C22—C21	0.74 (15)
C4—C17—C18—C19	179.87 (9)	C18—C19—C20—N2	178.09 (10)
C4—C17—C22—C21	−179.42 (9)	C18—C19—C20—C21	0.06 (15)
C5—C6—C7—C8	53.46 (11)	C19—C20—C21—C22	0.38 (15)
C5—C6—C7—C11	−67.23 (11)	C20—C21—C22—C17	−0.81 (16)
C5—C6—C7—C12	172.01 (9)	C22—C17—C18—C19	−0.29 (15)
C6—C5—C10—C4	−178.95 (8)	C23—N2—C20—C19	23.89 (15)
C6—C5—C10—C9	0.63 (13)	C23—N2—C20—C21	−158.17 (10)
C6—C7—C8—C9	−48.04 (11)	C24—N2—C20—C19	170.49 (10)
C7—C8—C9—N1	−160.24 (8)	C24—N2—C20—C21	−11.57 (15)
C7—C8—C9—C10	21.50 (14)		

### Hydrogen-bond geometry (Å, º)

**Table:**

*D*—H···*A*	*D*—H	H···*A*	*D*···*A*	*D*—H···*A*
N1—H1···O1^i^	0.879 (15)	1.968 (15)	2.8380 (11)	170.6 (14)
C13—H13*C*···O2^ii^	0.98	2.57	3.1741 (15)	120

Symmetry codes: (i) *x*−1/2, −*y*+1/2, *z*−1/2; (ii) −*x*, −*y*+1, −*z*+1.
